# Reducing Flavin and Ubiquinone Headgroups with Silicon Nanowire Photocathodes

**DOI:** 10.1021/aps.6c00003

**Published:** 2026-03-30

**Authors:** Elizabeth Lineberry, Andrew Liu, Nathan E. Soland, Wonseok Lee, Lihini Jayasinghe, Peidong Yang

**Affiliations:** † Department of Chemistry, 1439 University of California, Berkeley, Berkeley, California 94720, United States; ‡ Department of Materials Science and Engineering, University of California, Berkeley, Berkeley, California 94720, United States; § Chemical Sciences Division, Lawrence Berkeley National Laboratory, Berkeley, California 94720, United States; ∥ Materials Sciences Division, Lawrence Berkeley National Laboratory, Berkeley, California 94720, United States; ⊥ Kavli Energy Nanosciences Institute, Berkeley, California 94720, United States

**Keywords:** ubiquinone, riboflavin, photoelectrochemistry, photosynthetic biohybrid, silicon nanowire

## Abstract

Photosynthetic biohybrids—a structure composed of semiconducting electrodes and carbon dioxide-fixing autotrophs which can be energized by the electrode—offer a promising platform for selective CO_2_ reduction. However, studying the charge-transfer mechanisms from the semiconductor to the cell proves challenging due to a variety of simultaneous processes. Therefore, to deconvolute the system to understand photoelectrochemical performance, we employ model systems composed of a subset of the electron-transfer pathway. Here, we photoelectrochemically reduced ubiquinone-0 (UQ_0_) and riboflavin (Rf) (the head groups of ubiquinone-8/10 and flavin mononucleotide/flavin adenine dinucleotide) using Pt-decorated n^+^p-silicon nanowires, a robust catalytic architecture. Under irradiation with 100 mW cm^–2^ red light (740 nm), UQ_0_ and Rf were reduced with onset potentials of 0.876 V vs the reversible hydrogen electrode (V_RHE_) and 0.691 V_RHE_, respectively. In addition, UQ_0_ achieved a maximum Faradaic efficiency (FE) of 81% with a conversion rate of 1.22 μmol cm^–2^ h^–1^ at 0.75 V_RHE_, while Rf reached its maximum FE and rate at 73% and 0.167 μmol cm^–2^ h^–1^, respectively, at 0.55 V_RHE_. Both redox cofactors were continuously reduced over a 12 h period, demonstrating the robust photosynthetic biohybrid system.

## Graphical abstract

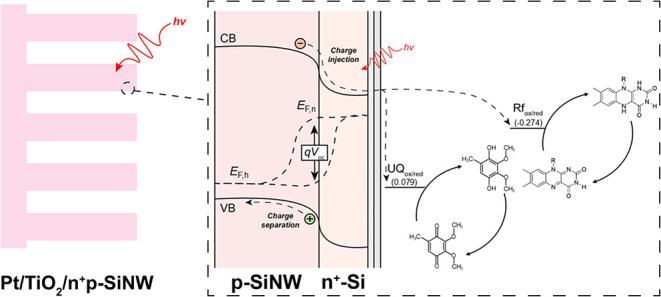

## Introduction

CO_2_-reducing photosynthetic biohybrid systems (PBS) are an efficient combination of light-harvesting semiconductors and acetogenic bacteria.^1–6^ The result is high Faradaic (electron utilization) efficiency toward acetate with near perfect carbon selectivity using the Wood-Ljungdahl Pathway (WLP).^7–9^ Semiconductor nanomaterials, such as silicon nanowires (SiNW), CdS, and gold nanoclusters, convert light energy to photoexcited electrons, which can serve as reducing agents for the CO_2_ reduction reaction, either through hydrogen produced by proton reduction, or via direct electron-transfer to the bacteria.^2,3,5,6,10,11^ SiNW are particularly useful for interfacing with bacteria, as they have many favorable photoelectrochemical (PEC) properties, including large catalytic surface area, benefiting reaction turnover, and broad solar absorption due to their low bandgap (∼1.1 eV) which improves light utilization efficiency.^12^ Additionally, they enable the close packing of bacteria, capitalizing on their high surface area.^13^

Charge flows from the semiconductor to the bacterium in two general pathways. The first process involves the semiconductor producing H_2_, which is absorbed into the cell. The electrons from the H_2_ enter the WLP through the action of the bifurcating hydrogenase, where they are used to reduce Ferredoxin (Fd) and nicotinamide adenine dinucleotide (NAD^+^) and subsequently enter the WLP.^9^ The second method to inject electrons into the WLP is non-hydrogen-mediated, or direct, electron-transfer through the membrane.^14^ Direct electron-transfer pathways are infrequently probed but of high interest to the biohybrid community, as they potentially offer opportunities to enhance charge-transfer rates within the cell and through the WLP, by suggesting targets for genetic engineering for example.^15^

However, the cell envelope is filled with numerous proteins and redox cofactors (Figure S1), which can complicate experimental attempts to monitor electron pathways through the cell. To facilitate study, the pathways can be subdivided, starting with the redox cofactors that shuttle electrons through the pathways’ proteins.^16–19^ To explore electron-transfer from SiNW to the cell, we can begin by investigating electron-transfer from the SiNW to important redox cofactors that comprise the electron transport chain, such as NAD^+^, Fd, cytochromes, flavins, and quinones.^7,9,14,16^ Understanding viable electron carrier interactions with the semiconductor component and the associated kinetics may enable the engineering or synthesis of biohybrids that have improved function.

In this study, we used this approach and examined the photoelectrochemical charge-transfer from SiNW to ubiquinone-0 (UQ_0_) and riboflavin (Rf), which act as models for UQ_8/10_ (present as a redox cofactor in the membrane of*Sporomusa ovata*­(*S. ovata*)) and flavin mononucleotide (FMN)/flavin adenine dinucleotide (FAD) (crucial in the active centers of numerous membrane-bound flavoproteins), respectively. These molecules contain the same redox centers as the cofactors they model, without the hydrophobic tail groups, as shown in Figure 1.

**Figure 1:**
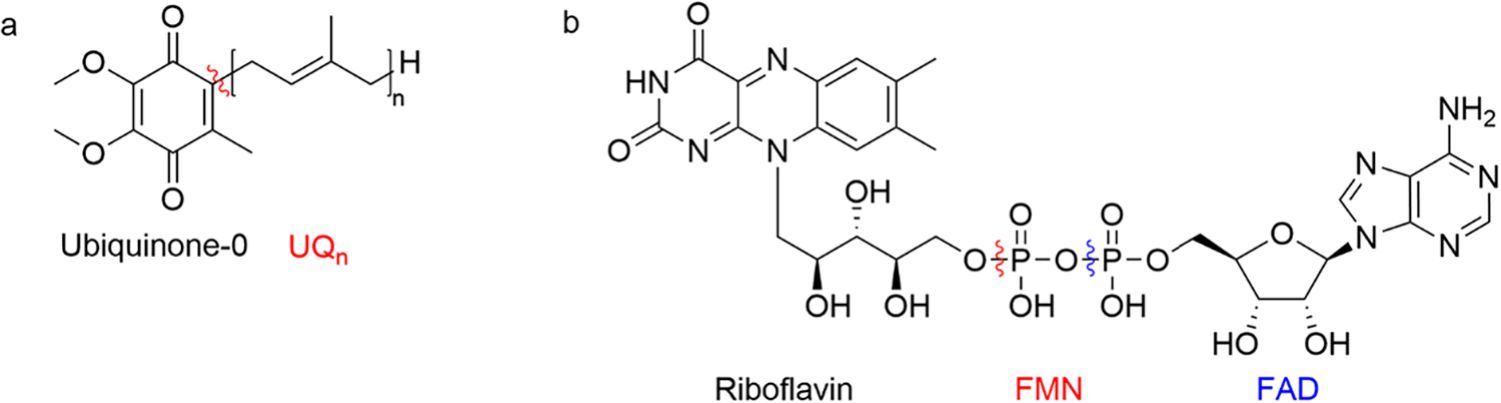
(a) The chemical structure of UQ_n_, with a red line designating the separating between the hydrophobic tail and the headgroup (UQ_0_). (b) The chemical structure of FAD with a blue line designating the difference between FAD and FMN, and a red line separating the headgroup (Rf).

As illustrated in Figure 2, we prepared an n^+^p-SiNW array with a protective TiO_2_ layer and decorated with Pt, as described in our prior biophotoelectrochemical (BPEC) experiments that used SiNW arrays to power*S. ovata*for CO_2_-to-acetate conversion,^4^ which had a typical photovoltage of 496 mV. Key photoelectrochemical metrics were gathered to indicate model efficacy. The reduction of UQ_0_ and Rf were individually monitored electrochemically under light conditions, with reduction onset potentials of 0.876 V vs the reversible hydrogen electrode (V_RHE_) and 0.691 V_RHE_, respectively. The Faradaic efficiencies (FE), a metric used to indicate electrochemical selectivity, and average conversion rates were 81.4% and 1.22 μmol cm^–2^ h^–1^, respectively, at 0.75 V_RHE_ for UQ_0_, and 72.6% and 0.167 μmol cm^–2^ h^–1^, respectively, at 0.55 V_RHE_ for Rf. To model the typical length of BPEC experiments and validate stability, the SiNW maintained consistent PEC reduction of both UQ_0_ and Rf for at least 12 h.

**Figure 2:**
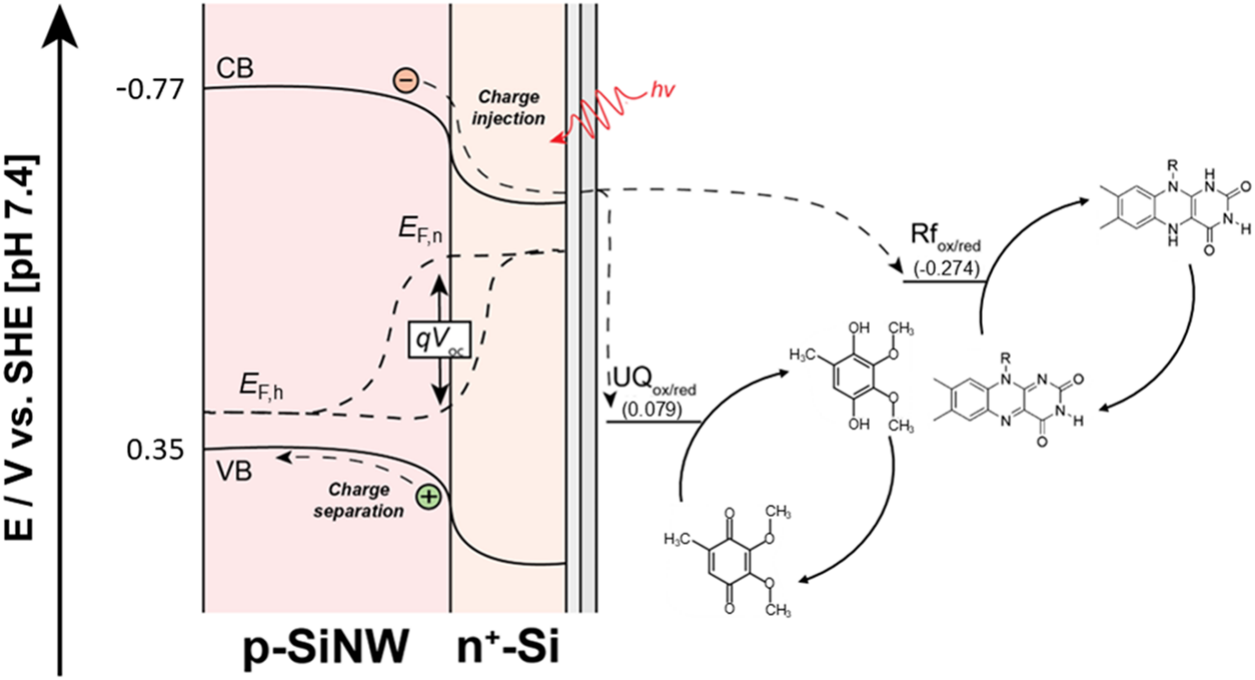
Illustration of Photocathodic UQ_0_ and Rf Reduction Driven by n^+^p-SiNW/TiO_2_/Pt. The photocathode absorbs red light to generate charge carriers and, from those, a photovoltage. Since the quasi-Fermi level of the electrons is more negative than the redox potentials of UQ_0_ and Rf, the photoexcited electrons are transferred to either UQ_0_ or Rf, along with two protons. *V*_ph_: photovoltage. CB: conduction band. VB: valence band. *E*_F,n_: quasi-Fermi level of electrons. *E*_F,h_: quasi-Fermi level of holes.

## Methods

### SiNW Fabrication

The p-SiNW array was made using photolithography and deep reactive ion etching. The radial n^+^p junction was formed by rapid thermal annealing with an arsenic silicate spin-on-dopant solution. Atomic layer deposition (ALD) was used to form a protective layer of TiO_2_ (approximately 10 nm). Pt was sputtered on the wires at 50 W for 30 s to deposit a 4 nm thick layer. More details can be found in the Supporting Information.

### PEC Experiments

PEC experiments were conducted with a Gamry Interface 1000 potentiostat using a three-electrode configuration. Experiments were conducted in a home-built, two-chamber electrochemical cell featuring a 0.375 cm^2^ contact area for the photocathode and Ag/AgCl reference electrode in the cathodic chamber, and a Pt wire counter electrode in the anodic chamber. The electrolyte was a Tris buffer solution (50 mM, pH 7.4) with N_2_ bubbled continuously through the catholyte. Cyclic voltammetry (CV) was performed until identical traces were obtained. All scans ran from the most positive potential in the cathodic direction and ended anodically at the starting potential. To determine UQ_0_ and Rf reduction onset potentials (*E*_onset_), the tangent lines of the non-Faradaic and Faradaic regions of a linear sweep voltammogram were calculated, and where the tangent lines converged indicated *E*_onset_. Power dependence was tested by adjusting the light distance. Photovoltage was measured using open-circuit potential (OCP) measurements. Chronoamperometry (CA) was performed at the desired potential for 1 h (or 12 h for stability experiments) under continuous stirring, and samples were taken before (after bubbling N_2_ in the catholyte for at least 20 min) and after the experiments.

### UQ_0_ and Rf Quantification

UQ_0_ was quantified using high-performance liquid chromatography (HPLC). The method was adapted from literature,^20^ and oxidized and reduced UQ_0_ were well-separated, eluting at 21.6 and 22.6 min, respectively, and quantitation was achieved by absorption and comparison to a calibration series. Ultraviolet–visible spectrophotometry (UV–vis) was used to quantify Rf. Since oxidized Rf has an absorbance peak at 445 nm that is not present in the reduced form,^21^ the decrease in the 445 nm peak was measured to determine how much oxidized riboflavin remained. For all experimental methods, no unexpected or unusually high safety hazards were encountered, and more details can be found in the Supporting Information.

## Results and Discussion

We implemented n^+^p-SiNW similar to previous work,^4,22^ coated with a TiO_2_ protection layer and decorated with Pt (Figure 3a,b). This design mimics the SiNW used for our biophotoelectrochemical system.^4^ The high contrast between the nanowires and the Si wafer underneath is due to the Pt layer.

**Figure 3:**
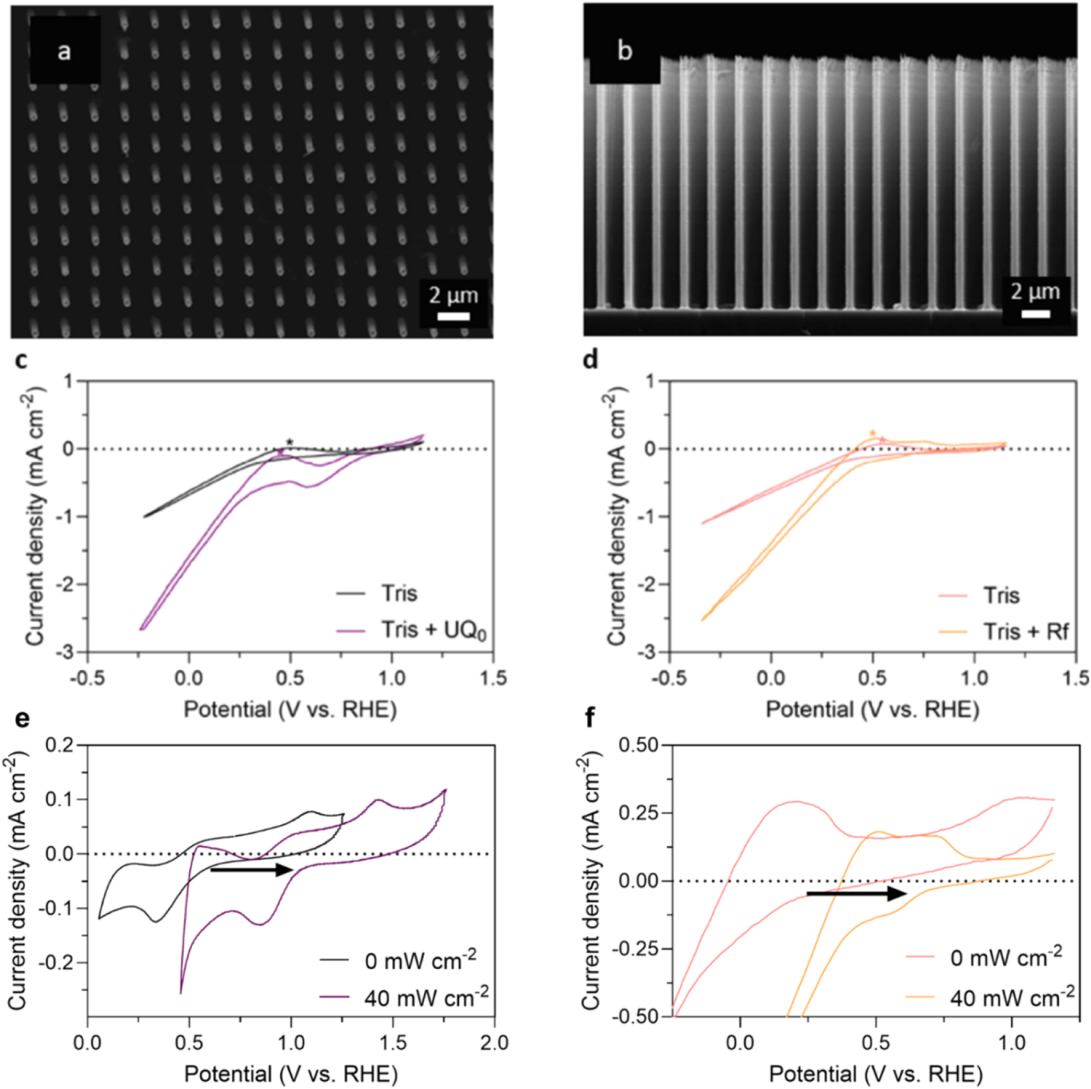
Reduction of UQ_0_ and Rf driven by SiNW photocathode. (a) Top-down and (b) cross-sectional scanning electron microscopic (SEM) images of SiNW electrode. (c) Cyclic voltammograms of SiNW with and without UQ_0_ (1 mM) and (d) Rf (0.5 mM) under 740 nm illumination. Scan rate: 100 mV s^–1^. The peaks labeled with an * are assigned to Pt–H desorption.^31^ (e) CVs of SiNW with UQ_0_ under 0 and 40 mW cm^–2^ illumination. Scan rate: 20 mV s^–1^. (f) CVs of SiNW with Rf under 0 and 40 mW cm^–2^ illumination. Scan rate: 100 mV s^–1^. Electrolyte solution: O_2_-depleted Tris buffer (50 mM, pH 7.4).

To explore the reduction of UQ_0_ (2,3-dimethoxy-5-methyl-1,4-benzoquinone) and Rf (which act as model molecules for the redox cofactors UQ_8/10_ and FMN/FAD, respectively), we investigated the photoelectrochemical redox behaviors of UQ_0_ and Rf on the n^+^p-SiNW/TiO_2_/Pt (SiNW) under red light (740 nm, 100 mW cm^–2^) in a two-compartment, three-electrode configuration, and electrochemically with a glassy carbon electrode (Figure S2). OCP measurements of the SiNW were conducted to determine their photovoltage (Figure S3). According to cyclic voltammetry (Figure 3c,3d), the SiNW exhibited the *E*_onset_ for UQ_0_ reduction at 0.876 V_RHE_ and an *E*_onset_ for Rf reduction at 0.691 V_RHE_, with a photovoltage of 496 mV, in an O_2_-depleted Tris buffer (50 mM, pH 7.4).

To investigate the photoelectrochemical system, we began by exploring the behavior of the Pt-SiNW system with cyclic voltammetry. The voltammetric behavior of both redox-active species resembles that expected from prior electrochemical studies on conventional electrodes.^23–31^ The UQ_0_ and Rf voltammograms each contain a single reduction peak, which (in buffered, aqueous electrolyte) involves the transfer of two electrons per molecule. Control experiments indicate that, within a restricted potential window excluding Pt oxidation, no reduction peaks are seen in CVs with no UQ_0_ (Figures 3c and S4a) or Rf (Figures 3d and S4b). UQ_0_ typically undergoes two electron-two proton reduction in buffered, aqueous media between pH 2–11 (Figure S5) to form the diol ubiquinol-0.^23,32^ As Pt acts as the catalyst in this system, there are several possible reaction pathways for UQ_0_. One pathway involves the 2-electron reduction of the redox molecule by SiNW, followed by the addition of 2 protons from water.^23,25–28^ Another proposed pathway is a concerted proton–electron transfer to the redox molecule.^29^ As the observed currents are a function of applied potential constructed from the combination of effects of background HER currents, UQ_0_ reduction, and a photovoltage generated under red light, UQ_0_’s voltammogram (Figure 3c) displays a net negative current in the anodic scan direction that lasts until the potential is reached at which UQ_0_ is no longer reduced (0.91 V vs RHE). Therefore, the exact origin of the negative current remains uncertain. The Pt–H desorption feature is superimposed (Figure S6) but not sufficient to generate a net positive current. The SiNW display identical redox behavior under dark conditions as well, provided one accounts for the photovoltage (Figure 3e). This behavior has also been seen on various Pt electrodes previously.^33^

Rf reduction likely undergoes a slightly distinct reduction process in this pH range. For Rf reduction, protonation does not happen directly at pH 7.4 and is postulated to undergo a two electron reduction in aqueous conditions before obtaining a proton to form the semiquinone species (Figure S5).^26^ Rf contains heterocyclic rings with nitrogen atoms that are each singly reduced (Figure 2). The 2-electron reduction cannot be kinetically resolved at scan rates of 5–1000 mV s^–1^ in an aqueous buffer, leading to only one apparent reduction peak in the CV.^23,30^ The Rf CV contains two oxidation peaks: one attributed to the Pt–H desorption peak, and one to the oxidation of reduced Rf (Figure 3d).^31^ Control experiments confirm that Pt–H desorption peaks are present in dark scans as well (Figures S6 and S7).

The free energy generated by an illuminated semiconductor can be quantified by its photovoltage, which is given by the splitting between the electron and hole quasi-Fermi levels that arises under illumination.^34^ We explored the power dependence of the SiNW photovoltage by increasing the light intensity from 0 mW cm^–2^ to 100 mW cm^–2^ (Figure 4a,b). There is significant photovoltage gain from 0 mW cm^–2^ to 20 mW cm^–2^, while there are milder enhancements from 20 to 100 mW cm^–2^. Under 20 mW cm^–2^, the SiNW achieve a photovoltage of around 425 mV, which nears 500 mV as the intensity increases to 100 mW cm^–2^. Increasing the illumination intensity amplifies the generation rate (and thus concentration) of photoexcited electrons (or holes), further separating the electron and hole quasi-Fermi levels. The electron and hole quasi-Fermi levels are related to the electron and hole concentrations (respectively) in a logarithmic fashion,^35^ leading to a nonlinear relationship between photovoltage and illumination intensity.

**Figure 4:**
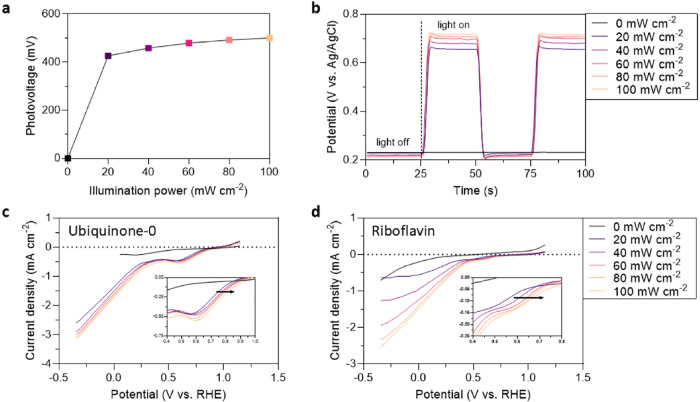
SiNW power dependence. (a) The effect of illumination power on overall photovoltage gain. (b) Open-circuit potential measurements of Pt-SiNW in Tris buffer at a 740 nm red light with intensities ranging from 0 to 100 mW cm^–2^. Linear sweep voltammograms of (c) UQ_0_ (1 mM) and (d) Rf (0.5 mM) at a 740 nm red light with intensities ranging from 0 to 100 mW cm^–2^. Electrolyte solution: O_2_-depleted Tris buffer (50 mM, pH 7.4). Scan rate: 100 mV s^–1^.

We investigated the resulting power dependence of UQ_0_ and Rf reduction (Figure 4c,4d) using linear sweep voltammetry to indicate activity changes in photoreduction as current magnitude at a range of potentials. For both UQ_0_ and Rf, the current of the hydrogen evolution reaction (HER), which begins after 0.5 V vs RHE in the cathodic scan, increases with the light intensity. There is no significant difference in current in the UQ_0_ and Rf reduction peaks; however, the onset potentials for UQ_0_ and Rf are both positively shifted due to the overall photovoltage gain. Due to this improvement, 100 mW cm^–2^ red light was used for photoelectrolysis to maximize yield.

After demonstrating charge-transfer from the SiNW to both UQ_0_ and Rf, we conducted bulk photoelectrolysis (chronoamperometry under irradiation) of UQ_0_ and Rf, which is particularly relevant to the biohybrid systems. To quantify reduction, we used HPLC to observe the transformation of UQ_0_ to its reduced form (ubiquinol-0). We observed the ubiquinol-0's peak at 22.6 min (Figure S8). For Rf quantification, we utilized UV–vis to monitor the decrease in the peak at 445 nm, signifying the reduction of Rf (Figure S9). Figure S10 shows the increase of UQ_0_’s reduction peak on HPLC and the decrease of Rf’s oxidation peak on UV–vis over a 12-h period of photoelectrolysis. To demonstrate the necessity of photoelectrochemical reduction, controls with and without light and electrical bias were run. In the presence of both light and applied bias, the SiNW reduced UQ_0_ with a FE of 81.4% and a conversion rate of 1.22 μmol cm^–2^ h^–1^ (100 mW cm^–2^ red light, 0.75 V_RHE_), and Rf with a FE of 72.6% and a conversion rate of 0.167 μmol cm^–2^ h^–1^ (100 mW cm^–2^ red light, 0.55 V_RHE_) in O_2_-depleted Tris buffer (27 mL, 50 mM, pH 7.4). If either light was removed at the same applied potential, or electrical bias was removed, no UQ_0_ or Rf was reduced (Figure 5b,5d). Without bias, water splitting cannot occur, since Si’s 1.1 eV bandgap is not thermodynamically sufficient for water splitting (1.23 V).^36^ Meanwhile, dark conditions lead to a −0.496 V shift in photoelectrochemical bias, such that for the same applied electrochemical bias, the SiNW are no longer sufficiently reducing. These results emphasize the importance of high photovoltage to achieve the mild applied bias.

**Figure 5:**
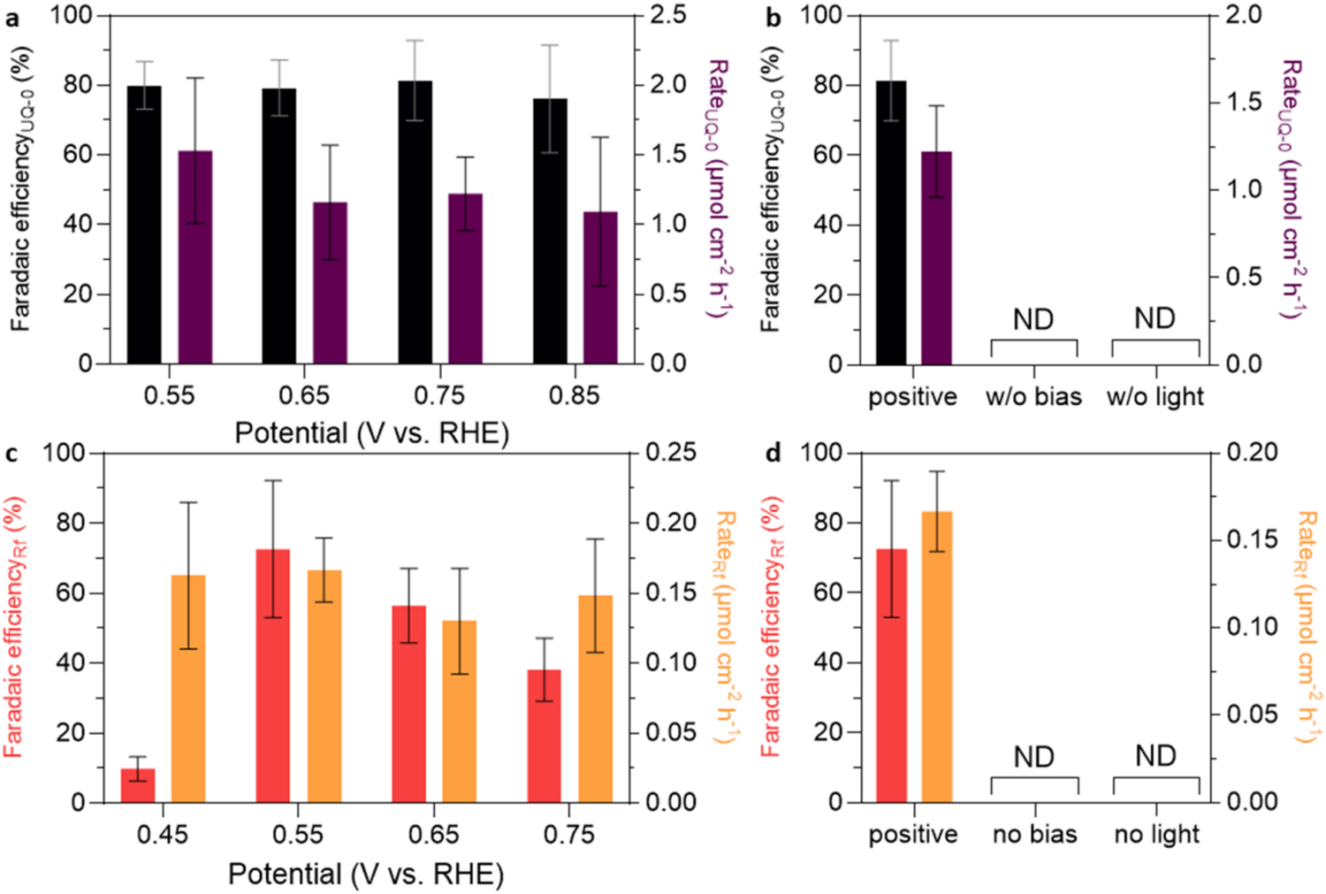
SiNW-driven reduction of UQ_0_ and Rf. (a) Influence of potential on UQ_0_ reduction, and (b) control experiments for photocatalytic UQ_0_ reduction in 0.5 mM UQ_0_ (w/o light control run at 0.75 V_RHE_). (c) Influence of potential on Rf reduction, and (d) control experiments for photocatalytic Rf reduction in 50 μM Rf (w/o light control run at 0.55 V_RHE_). Electrolyte in (a–d): O_2_-depleted Tris buffer (27 mL, 50 mM, pH 7.4), continuously stirred. Light intensity in (a–d): red light (740 nm, 100 mW cm^–2^). ND: not detected.

Next, we tested the effect that applied potential has on the FEs and conversion rates of UQ_0_ and Rf reduction. UQ_0_ reduction reached a maximum FE at 0.75 V_RHE_ (Figure 5a). The FE stays relatively constant from 0.55 to 0.85 V_RHE_, while the rate increases with the current. This confirms that UQ_0_ is preferentially reduced at those potentials, where HER is not expected to occur, and the nonunity FE is likely caused by oxidation during sample transfer, as quinols are readily oxidized in the presence of O_2_,^37^ thus leading to a likely underestimation of the actual efficiency. Rf reduction reached a maximum rate and FE at 0.55 V_RHE_ (Figure 5c). It is well-known that Pt-decorated SiNW efficiently perform HER in aqueous environments.^38^ Since the photovoltage of the SiNW is over 450 mV, they begin performing HER at 0.45 V_RHE_. The FE of Rf reduction drops as HER takes over; however, the rate remains relatively constant, which could imply a diffusion-limited process for Rf reduction. Thus, the lower formal reduction potential of Rf leads to a greater likelihood of competition with HER compared to UQ_0_ as reducing power is increased within the range that the biohybrid PEC system typically operates.

Finally, we investigated the stability of the SiNW photocathode under red light (740 nm, 100 mW cm^–2^) in N_2_-purged Tris buffer (27 mL, 50 mM, pH 7.4) with the optimal bias for each mediator. As shown in Figure 6a,6c, UQ_0_ was consistently reduced over a 12-h period. The current remained stable throughout the experiment, with the decrease commensurate with the decreasing amount of oxidized UQ_0_ available (Figure S11), and the morphology of the SiNW had negligible changes (Figure S12). Rf was also stably reduced for 12 h, as demonstrated in Figure 6b,6d. The SiNW delivered stable current to the Rf throughout the 12 h (Figure S13), and negligible structural changes were seen in the SiNW after the 12 h (Figure 6e,f). These results demonstrate the robustness of the nanowires, which is a desirable trait, especially for the long-term microbial growth and operation required of BPEC systems.^13,39^ The temperature change under illumination was recorded to rule out thermal effects on reduction (Figure S14). The catholyte started at 21.8 °C before increasing sharply to 26 °C in the first hour, then stayed consistently around 29 °C for the remainder of the experiment starting at 3 h, while the reduction rate remained consistent before and after temperature stabilization.

**Figure 6:**
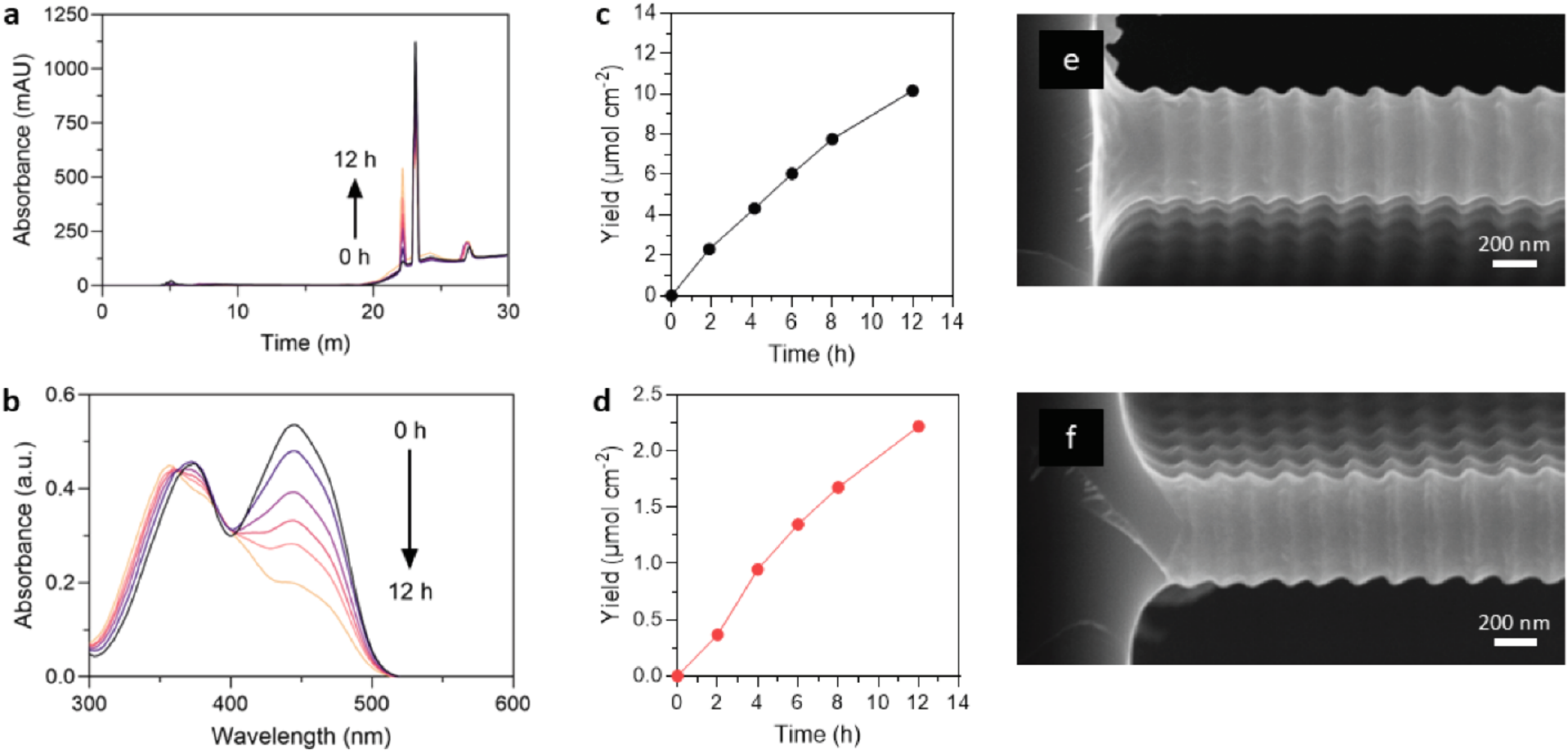
Controlled potential photoelectrolysis for UQ_0_ and Rf reduction. (a) HPLC spectra of UQ_0_ reduction (0.5 mM UQ_0_ in an O_2_-depleted Tris buffer (27 mL, 50 mM, pH 7.4), continuously stirred. Applied potential: 0.75 V_RHE_) and (b) UV–vis spectra of Rf reduction (50 μM Rf in an O_2_-depleted Tris buffer (27 mL, 50 mM, pH 7.4), continuously stirred. Applied potential: 0.55 V_RHE_), and time profiles of (c) UQ_0_ and (d) Rf, over a 12-h period. Cross-sectional SEM images of SiNW (e) before and (f) after photoelectrolysis (Rf).

This study is the first demonstration of direct photoelectrochemical reduction of UQ_0_ and Rf by a SiNW photocathode. This is an important step in exploring the fundamentals of direct electron injection into the electron transport pathways via known redox cofactors in photosynthetic biohybrids. Follow-up studies should aim to further examine, with photoelectrochemical and spectroscopic methods, the crucial redox cofactors required for direct electron-transfer and electron-transfer in the WLP. The fundamental redox cofactors, such as those employed in this study, are ubiquitous in all redox pathways, and (photo)­electrochemical information forms the basis for further model systems and exploration of electron-transfer through photosynthetic biohybrids. For instance, cytochromes,^40^ NAD­(P)^+^,^41^ and Ferredoxin^42^ are crucial electron shuttles, whose redox potentials are shown in Figure 7, as well as UQ_8/10_ ^43^ and FMN/FAD,^44^ the molecular analogues studied here. Each of these redox cofactors falls within the bandgaps of Si and CdS (Figure 7), two common semiconductors for PBS,^2,45^ and should be further explored under BPEC and biophotochemical conditions to improve understanding of fundamental photoelectrochemical kinetics for future optimizations.

**Figure 7:**
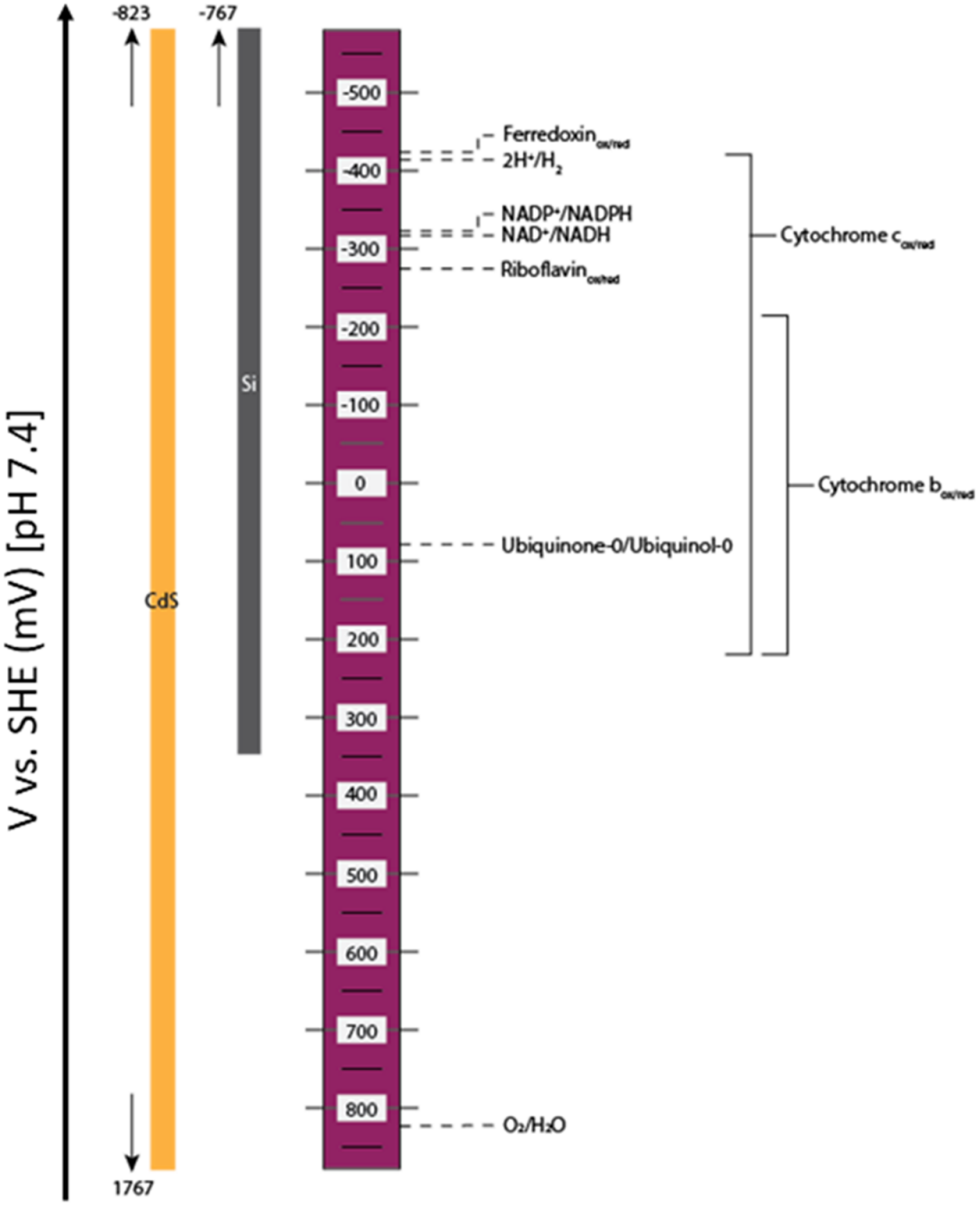
Redox potentials of important redox cofactors in *S. ovata* and semiconductors for PBS. Standard redox potentials and ranges are indicated with dashed lines on the right side of the plot. The band gaps of CdS and Si at pH 7.4 are shown on the left side. b- and c-type cytochromes cover a wide range of potentials, shown with solid brackets. *S. ovata* and other acetogens do not contain all cytochromes covering this range.^46^

## Conclusion

In this study, we achieved the photoelectrochemical reduction of UQ_0_ and Rf using silicon nanowire electrodes. Using 100 mW cm^–2^ red light, the SiNW displayed a photovoltage of 496 mV, leading UQ_0_ to achieve the highest FE at 0.75 V_RHE_ and Rf at 0.55 V_RHE_. UQ_0_ was reduced with a maximum FE of 81.4% and a rate of 1.22 μmol cm^–2^ h^–1^, while Rf was reduced with a maximum FE and rate of 72.6% and 0.167 μmol cm^–2^ h^–1^. Furthermore, the SiNW demonstrated stable performance for at least 12 h, continuously reducing the redox cofactors with stable current and negligible morphological changes. This model system demonstrated the suitability of SiNW for direct electron-transfer in PBS, thereby expanding the knowledge base of redox cofactor PEC in environments that mimic BPEC conditions.

## Supplementary Material



## Notes

Additional experimental details of photocathode preparation, photoelectrochemical analysis, quantification of UQ0 and Rf, scanning electron microscopy (SEM) characterization; Sporomusa ovata cell envelope schematic, supporting cyclic voltammograms, open-circuit voltage data, UQ0 HPLC chromatograms, Rf UV–Vis spectra, chronoamperomograms, additional SEM images, and temperature time profile (PDF)
