# Supplementary material for: Reducing Flavin and Ubiquinone Headgroups with Silicon Nanowire Photocathodes
Source: Artif Photosynth. 2026 Mar 30;2(4):234–42. doi: 10.1021/aps.6c00003 (PMC13403228; doi:10.1021/aps.6c00003)
Supplement: Supplementary file 1 [file af6c00003_si_001.pdf]

Supporting Information

Reducing Flavin and Ubiquinone Headgroups with Silicon Nanowire Photocathode

Elizabeth Lineberry,[1] Andrew Liu,[2] Nathan E Soland,[1] Wonseok Lee,[2] Lihini Jayasinghe,[1] and Peidong Yang[1,2,3,4,5,*]

[1]Department of Chemistry, University of California, Berkeley, Berkeley, CA 94720, USA

[2]Department of Materials Science and Engineering, University of California, Berkeley, Berkeley, CA 94720, USA

[3]Chemical Sciences Division, Lawrence Berkeley National Laboratory, Berkeley, CA 94720, USA

[4]Materials Sciences Division, Lawrence Berkeley National Laboratory, Berkeley, CA 94720, USA

[5]Kavli Energy Nanosciences Institute, Berkeley, CA 94720, USA

[*]E-mail: p_yang@berkeley.edu

Supplementary Methods

Chemicals and Materials.

2,3-dimethoxy-5-methyl-1,4-benzoquinone (Ubiquinone-0), Riboflavin, and Tris buffer were purchased from Sigma-Aldrich (St. Louis, MO). 2,3-dimethoxy-5-methyl-1,4-hydroquinone was purchased from Biosynth International Inc. (Staad, Switzerland). All chemicals were used without further purification. We used type 1 ultrapure water (18.2 MΩ cm) from a Direct-Q® 5 UV ultrapure water purification system (Millipore Corp., USA).

Preparation of n^+p-type silicon nanowire photocathodes.

We synthesized n^+p-type silicon nanowire (n^+p-SiNW) according to the literature (*J. Am. Chem. Soc*. **2023**, *145*, 12987−12991). We used p-type boron-doped 6” wafers (<100> oriented, 10-30 Ω cm). Using a typical photoresist process, we applied hexamethyldisilazane for 2 minutes and spin-coated on Dow UV210GS-0.6 photoresist to have a thickness of 0.87 μm. Using a 4x deep UV photolithography stepper (ASML DUV Stepper Model 5500/300) with a mask that is patterned with a square lattice of 3 μm circles and pitch of 8 μm, we patterned this for final

resist pattern of 0.75 μm circles and 2 μm pitch. This was developed using MF-26A for 45 seconds, descummed with O_2 plasma at 50 W for 60 seconds, then hard baked at 140 °C with UV light. To etch the wafer, we used a low-frequency inductive-coupled plasma deep reactive ion etch (DRIE) process (Surface Technology Systems Advanced Silicon Etch) using O_2 and SF_6 as the etch gas, C_4F_8 as the passivation gas, and a typical DRIE smooth-wall recipe until nanowire lengths of 21 μm and diameters of 550 nm were achieved. Afterwards, we removed any remaining photoresist with O_2 plasma at 400 W for 2.5 minutes.

We spin-coated the carrier wafer with an arsenic silicate spin-on-dopant solution (Filmtronics, Inc.) at 2200 rpm for 30 seconds and baked it on a hotplate at 150 °C for 30 minutes. We then cleaned the nanowire arrays with HF, water, and acetone right before gently placing them onto the carrier wafer such that the nanowire arrays were touching the dopant layer and placed the wafers into a rapid thermal annealing chamber at 900 °C for 3 minutes under N_2.

We cleaned the n^+p-SiNW substrate in a 16% HF bath for 3 minutes and thoroughly washed with DI water and acetone and then dried. Then, a 10 nm TiO_2 layer was deposited at 200 °C using atomic layer deposition and tetrakis(dimethylamido)titanium as the precursor (Cambridge Fiji 200 Plasma ALD system).

We deposited the Pt catalyst using a multi-target co-sputtering system (built in-house) with 3x 3” TORUS Mag Keeper sputter guns, supplied by two 2 kW pulsed DC power supplies and one 1.5 kW DC power supply. Around 4 nm of Pt catalyst was sputtered onto the SiNW substrate with 30 seconds of 50 W power applied on the Pt target after TiO_2 deposition. The Pt/SiNW were stored in ambient air until use.

To fabricate the electrode, we scratched Ga-In eutectic onto the back of the substrate and applied quick drying silver paint on top of the scratched surface, then used double-sided conductive carbon tape to fix the chip onto Ti foil to create an ohmic contact. Following this, we sealed the sides and back of the Si nanowire photocathode with nail polish, allowed the electrode to dry for 30 minutes and mounted the electrode to the cell for photoelectrochemical measurements.

Photoelectrochemical analysis.

As previously described (*J. Am. Chem. Soc.* **2023**, *145* (36), 19508-19512), we used a Gamry Interface 1000 potentiostat (Gamry Instrument, Warminster, PA) to conduct (photo)electrochemical experiments in a three-electrode configuration. A photocathode was mounted on the back of a home-built electrochemical cell with a contact area of 0.375 cm^2 and sealed with an X-ring, and the reference electrode (Ag/AgCl, 1 M KCl, CH Instruments, Kwun Tong, Hong Kong) was placed in a cathodic chamber. Separated by an anion exchange membrane (AMI-7001S, Membranes International, Ringwood, NJ), a Pt wire counter electrode was placed in an anodic chamber. The electrolyte was a Tris buffer solution (50 mM, pH 7.4), and each chamber contained 30 mL. N_2 was continuously bubbled through the catholyte during measurements. To conduct photoelectrochemical experiments, we used a 740 nm LED, calibrating the LED with a Si photodiode (Thorlabs FDS1010-CAL) to ensure that an intensity of 100 mW cm^{-2} was reaching the photocathode. We purged the cell with N_2 for at least 20 minutes before running any measurements. Cyclic voltammetry (CV) was performed to test the performance of the photocathode before adding the redox cofactor (either ubiquinone-0 (UQ_0) or riboflavin (Rf)). We added the redox cofactor and ran CV (100 mV s^{-1}). Then, chronoamperometry was run at the desired potential. All potentials were converted to vs. RHE according to equation S1:

$$E_{\mathrm{RHE}}\ (\mathrm{V}) = E_{\mathrm{Ag/AgCl}}\ (\mathrm{V}) + 0.222 + (0.059 \times \mathrm{pH}) \quad \text{(S1)}$$

To determine the onset potential (E_{onset}), we calculated the tangent lines of the non-Faradaic and Faradaic regions of the CV and found where the tangent lines converged (*Curr. Opin. Electrochem*. **2023**, *37*, 101176; *Nature Chem.* **2018**, *10*, 24–30). This potential indicated E_{onset}. We estimated the photovoltage (V_{ph}) of a photocathode with open circuit potential measurements in light versus dark conditions.

Quantification of UQ_0 and Rf.

Quantification of UQ_0 was carried out through high performance liquid chromatography (HPLC). Adapted from literature (*Bioelectrochemistry.* **2016**, *111*, 100–108), we used a C18 column with dimensions 4.6 × 100 mm, particle diameter 2.7 µm (Agilent, U.S.). The mobile phase was a mixture of water and methanol, which began at 5% (*V/V*) methanol, reached 10% at 10 min, 85% at 15 min, and 100% methanol at 25 min, and ran for a total of 30 min. The injection volume was 10 µL, the column temperature was 40 °C, and the flow rate was 0.2 mL min^{-1}. The HPLC system was equipped with a G7111B quaternary pump, a G1313A autosampler, and a G7165A multiple wavelength detector, controlled by ChemStation software (Agilent). The chromatograms were recorded at 210 nm. The hydroquinone eluted at 21.6 min, and the quinone eluted at 22.6 min (**Figure S3**). A standard curve was developed with a 2,3-dimethoxy-5-methyl-1,4-hydroquinone standard measured at 0.25 mM, 0.5 mM, and 1 mM.

Quantification of Rf was carried out through UV-Vis spectrophotometry. According to literature (Structure and General Properties of Flavins. In *Flavins and Flavoproteins: Methods and Protocols*; Springer, 2014; pp 3–13), there is a peak at 445 nm that is present for oxidized Rf but is not present in the reduced Rf spectrum. The instrument was a UV-2600 UV-Vis Spectrophotometer (Shimadzu, Kyoto, Japan). The oxidized Rf standard was measured at 12.5

μM, 25 μM, and 50 μM to prepare a standard curve. Since all experiments were carried out under 740 nm light, which cannot photo-degrade Rf to lumichrome (*Chem. Phys.* **2005**, *308*, 69–78), the concentration of reduced Rf was determined by subtracting the final Rf_{ox} concentration for the starting concentration.

We calculated the Faradaic efficiency (FE) with equation S2:

$$\mathrm{FE_{NADH}} = \frac{96485 \times 2 \times \text{moles of } \mathrm{UQ}_{0,red} \text{ or } \mathrm{Rf}_{red}}{\int I dt} \quad \text{(S2)}$$

where *I* is the current and *t* is the reaction time.

Scanning electron microscopic (SEM) characterization.

We imaged the SiNWs before and after photoelectrolysis with SEM at 10 kV by Ultra 55 SEM (ZEISS Microscopy, Jena, Germany). Following electrolysis, we rinsed the photocathode with DI water and acetone, drying thoroughly before SEM characterization. The photocathode was cleaved in half to characterize the cross section in the center of the electrode.

Supplementary Figures

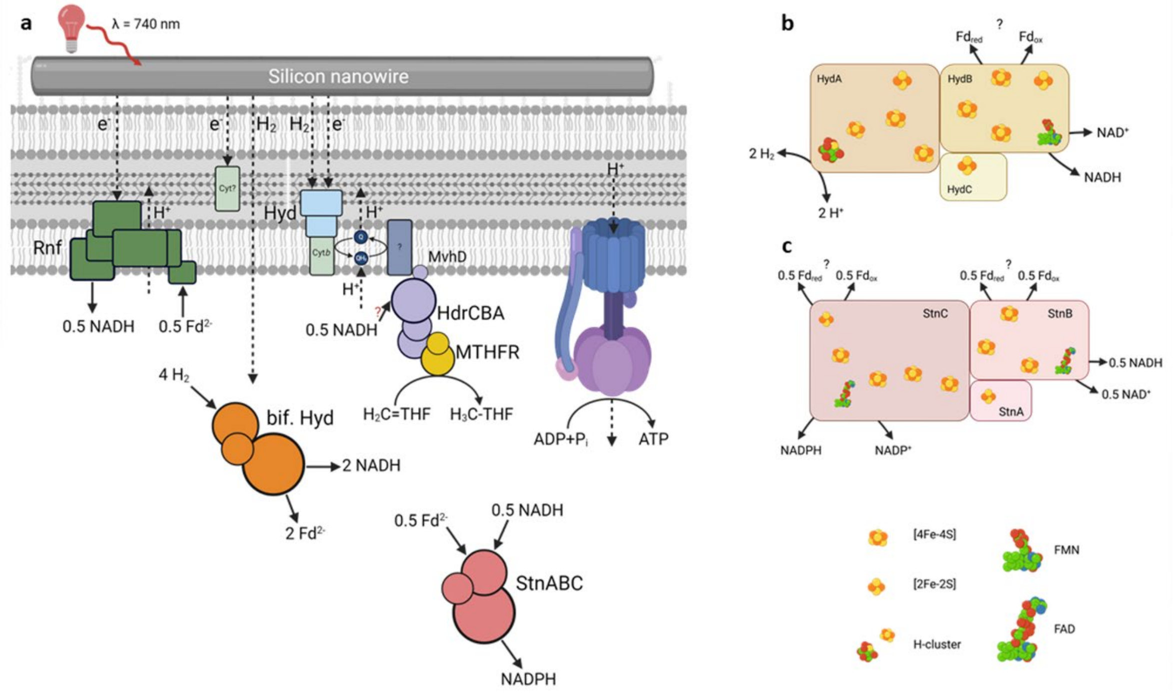

Figure S1. Schematic illustration of *S. ovata* electron transport chains and possible interactions with the photocathode. (a) Possible electron transport mechanisms in *S. ovata* via hydrogenases and membrane-bound cytochromes. Depictions of bif. Hyd. (b) and Stn. (c) with electron carriers. Hyd: membrane-bound hydrogenase. bif. Hyd: electron-bifurcating hydrogenase. Cyt*b*: cytochrome b. Q/QH_2: ubiquinone/ubiquinol. StnABC: *Sporomusa*-type NADH-dependent reduced ferredoxin:NADP+ oxidoreductase. Cyt? represents a possible cytochrome c in the peptidoglycan layer that could help facilitate charge transfer.

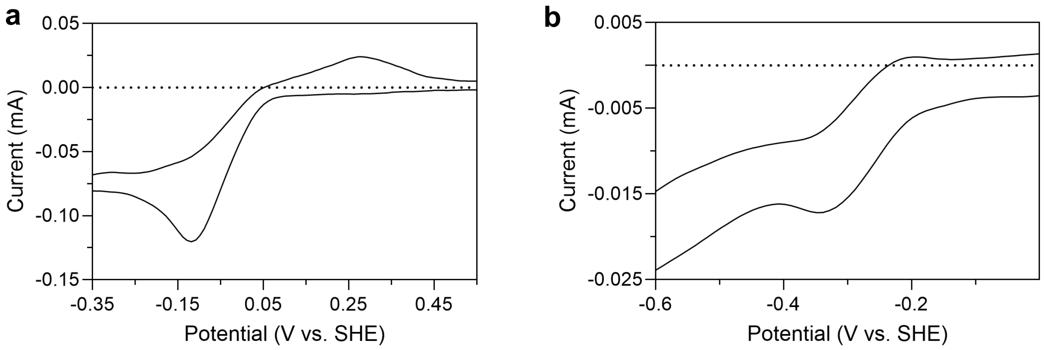

Figure S2. Cyclic voltammograms of UQ_0 (a) and Rf (b) on a glassy carbon electrode. The wider peak separation for UQ_0 is consistent with typical ubiquinone behavior in this pH range. Kinetic hinderance of electron transfer or protonation transfer, leading to the peak separation, is observed for UQ_0 between pH 2.5 to 10.5 (*J Solid State Electrochem.* (2016) 20:3229–3238). Scan rate: 100 mV s^{-1}. Electrolyte solution: O_2-depleted Tris buffer (50 mM, pH 7.4).

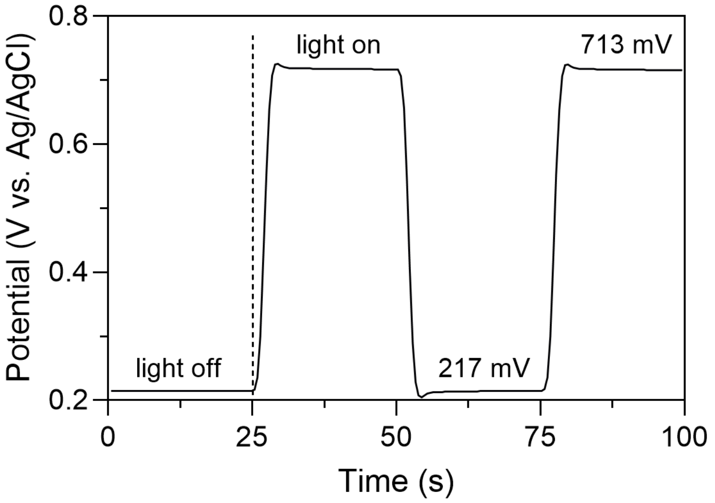

Figure S3. Open circuit voltammogram of SiNW under light and dark conditions in the absence of UQ_0/Rf. Light intensity: 100 mW cm^{-2} red light. Electrolyte solution: O_2-depleted Tris buffer (50 mM, pH 7.4).

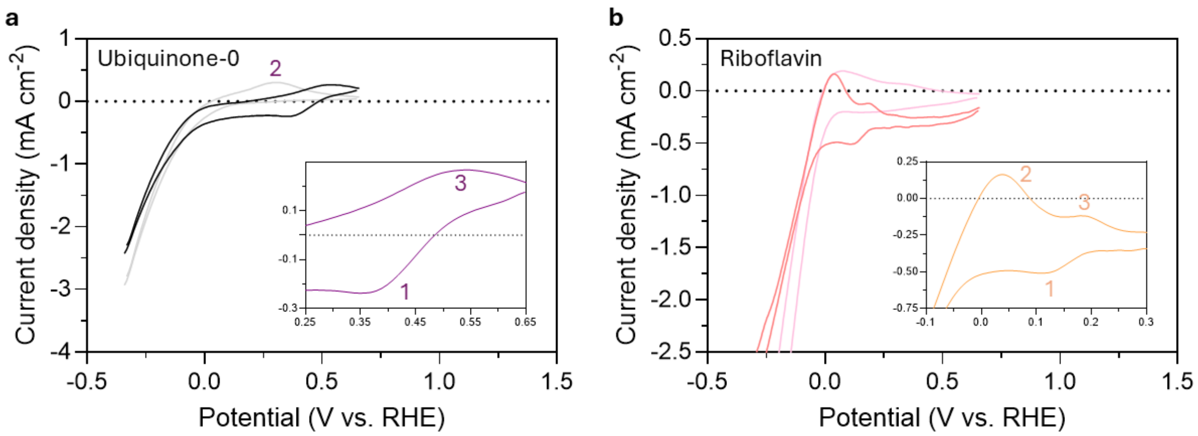

Figure S4. Cyclic voltammograms of Pt foil for (a) UQ_0 (0.5 mM) and (b) Rf (0.5 mM). The lighter CVs in the background are blank controls that do not contain UQ_0/Rf. Electrolyte solution: O_2-depleted Tris buffer (50 mM, pH 7.4). Light intensity: 100 mW cm^{-2} red light. Scan rate: 100 mV s^{-1}. The peaks labeled 1 are assigned to the 2-electron reduction of Rf/UQ_0, peaks labeled 2 are assigned to Pt-H desorption, and the peaks labeled 3 are assigned to the 2-electron oxidation of Rf/UQ_0 (*Proc. Natl. Acad. Sci.* **2009**, *106* (43), 18143–18148). Peak 2 is not seen in the CV containing UQ_0 due to the saturation of UQ_0 on the Pt surface. As the electrode becomes saturated with the UQ_0 film, the hydrogen desorption peak is suppressed (*J. Phys. Chem*. **1984**, *88*, 4583-4586).

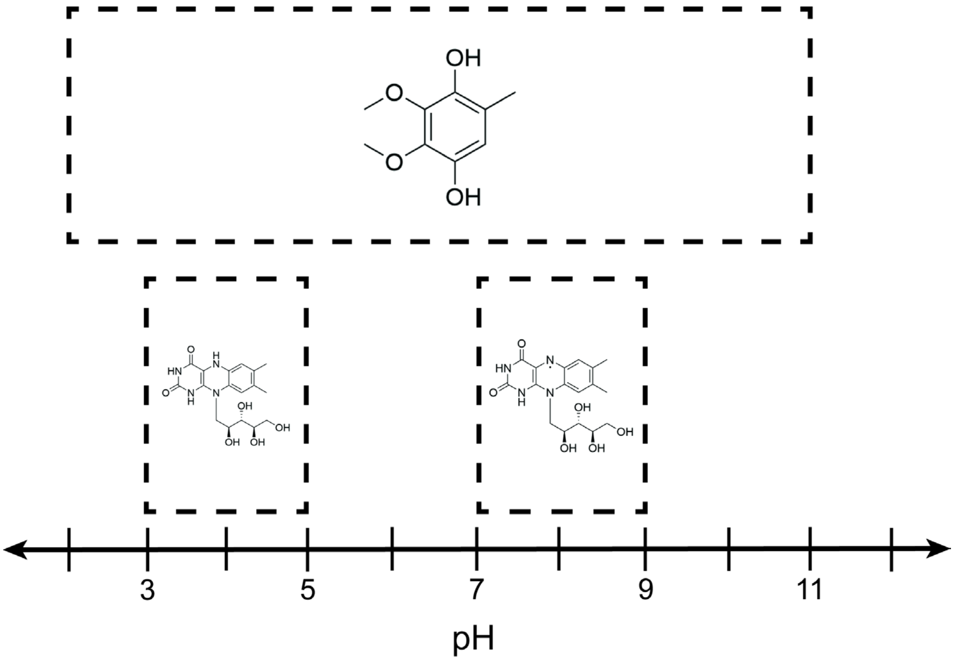

Figure S5. Reduction products of UQ_0 and Rf. Ubiquinol is fully protonated from pH 2-11 (*J. Am. Chem. Soc.* **2007**, *129* (42), 12847–12856; *J. Solid State Electrochem.* **2016**, *20* (12), 3229–3238). Rf is fully protonated from pH 3-5, but Rf is only partially protonated (2 e^-, 1 H^+ reduction) in the 7-9 pH range (*J. Phys. Chem. B* **2013**, *117* (44), 13755–13766).

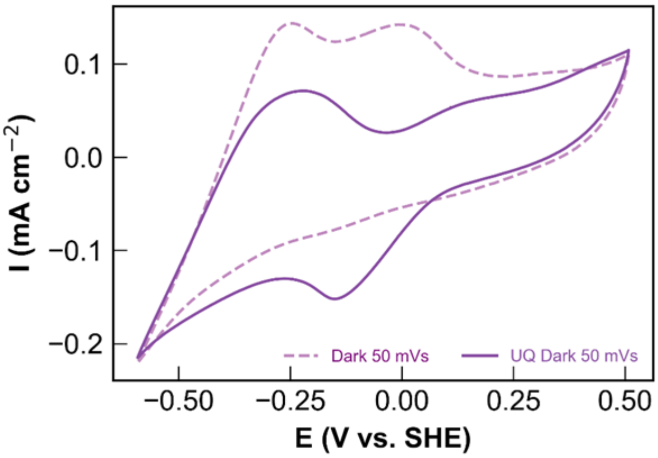

Figure S6. CVs of n$^+$p-SiNW/TiO_2/Pt with and without UQ_0 (0.5 mM) in the absence of light. Scan rate: 50 mV s^{-1}. Electrolyte solution: O_2-depleted Tris buffer (50 mM, pH 7.4).

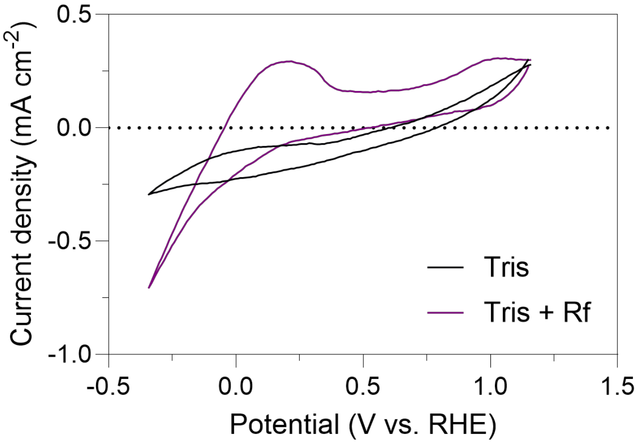

Figure S7. CVs of n^+p-SiNW/TiO_2/Pt with and without Rf (0.5 mM) in the absence of light. Scan rate: 100 mV s^{-1}. Electrolyte solution: O_2-depleted Tris buffer (50 mM, pH 7.4).

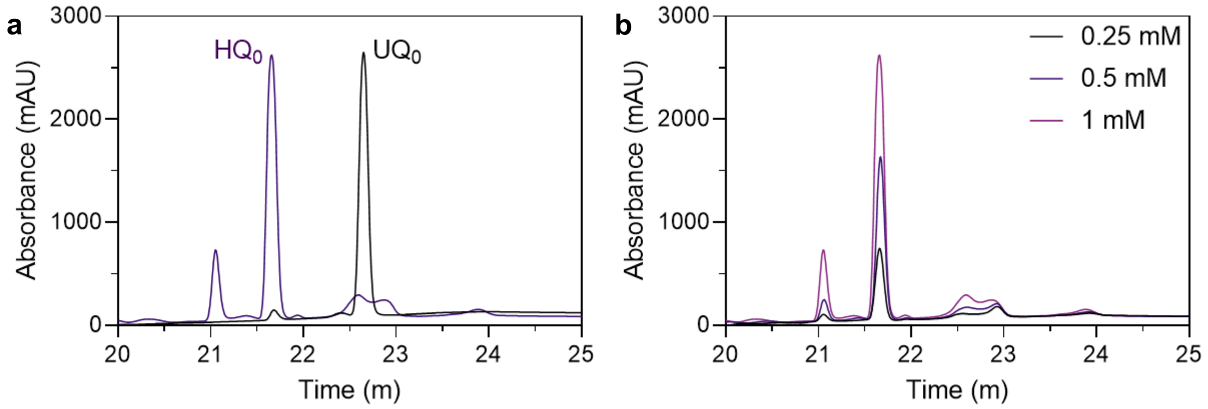

Figure S8. HPLC chromatograms of ubiquinol/hydroquinone (HQ_0) and oxidized UQ_0 (a) and 0.25 mM, 0.5 mM, and 1 mM HQ_0 (b).

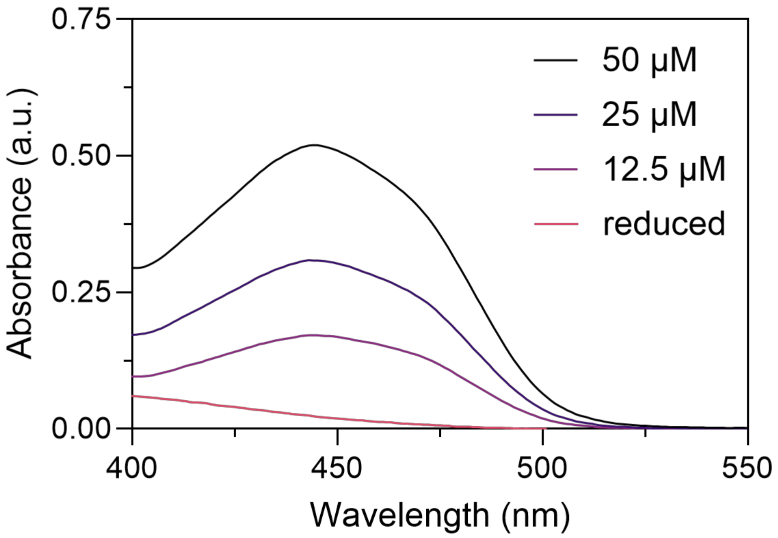

Figure S9. UV-Vis spectra of 50 μM, 25 μM, and 12.5 μM oxidized Rf, and 50 μM reduced Rf. The decrease in 445 nm absorbance was used to calculate the decrease in oxidized Rf and determine the concentration of reduced Rf.

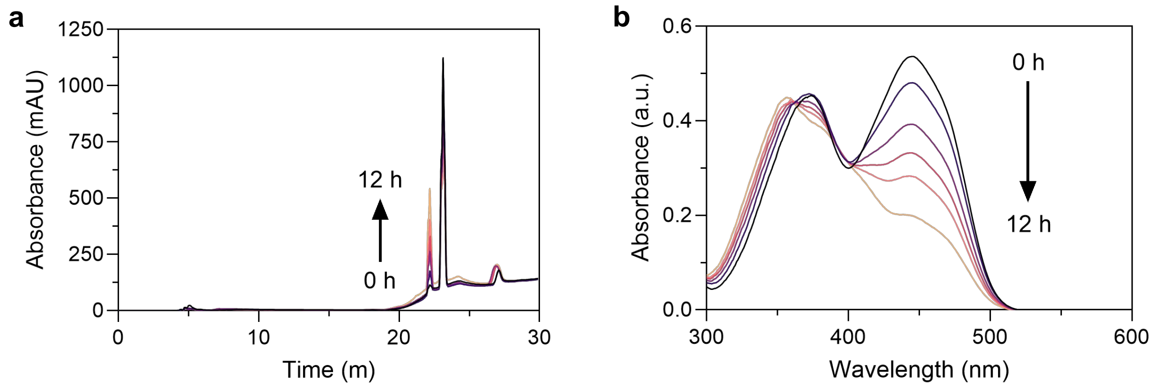

Figure S10. HPLC chromatograms of UQ_0 (a) and UV-Vis spectra of Rf (b) over 12 h. UQ_0 experimental parameters: 0.5 mM UQ_0 in an O_2-depleted Tris buffer (27 mL, 50 mM, pH 7.4), continuously stirred; applied potential: 0.75 V_{RHE}. Rf experimental parameters: 50 µM Rf in an O_2-depleted Tris buffer (27 mL, 50 mM, pH 7.4), continuously stirred; applied potential: 0.55 V_{RHE}.

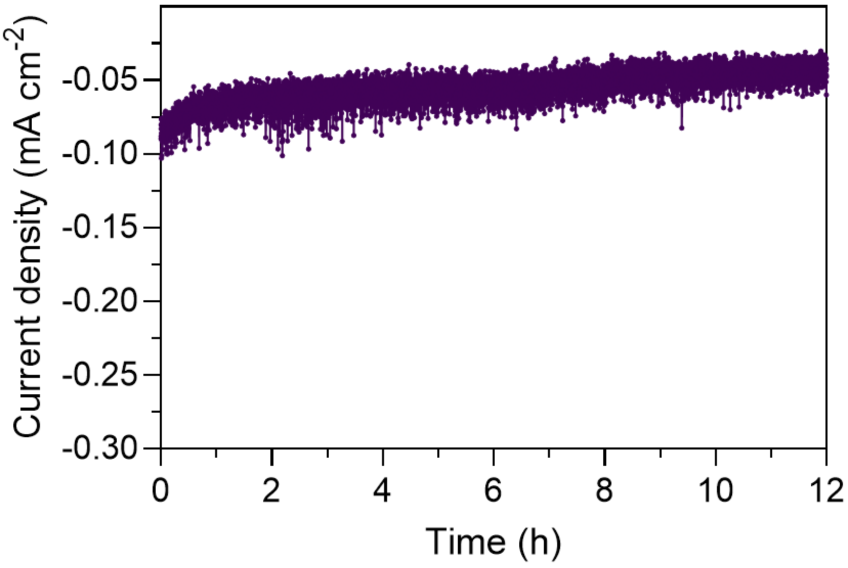

Figure S11. Chronoamperometry (CA) of UQ_0 (0.5 mM) reduction over 12 hours at 0.75 V_{RHE}. Light intensity: 100 mW cm^{-2} red light. Electrolyte solution: O_2-depleted Tris buffer (50 mM, pH 7.4).

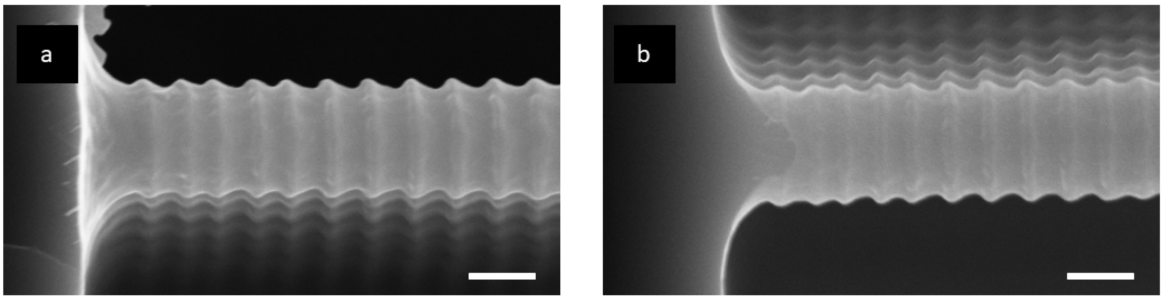

Figure S12. Cross-sectional scanning electron microscopic images of SiNW before (a) and after (b) 12-h UQ_0 reduction. Scale bars: 300 nm.

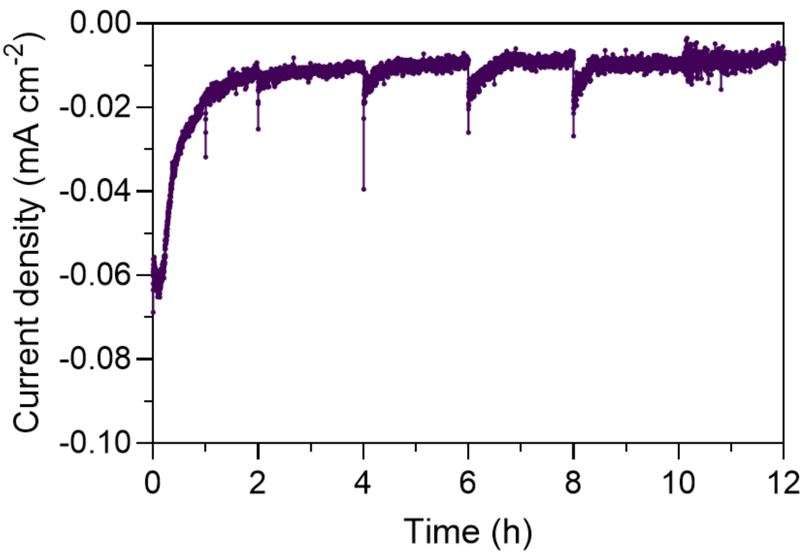

Figure S13. CA of Rf (50 µM) reduction over 12 hours at 0.55 V_{RHE}. Light intensity: 100 mW cm^{-2} red light. Electrolyte solution: O_2-depleted Tris buffer (50 mM, pH 7.4). Interruptions in the scan are due to sample collection for quantification.

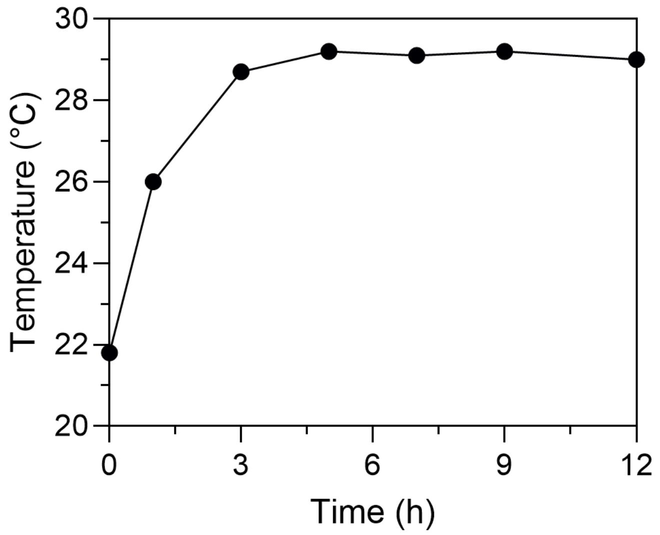

Figure S14. Catholyte temperature change during UQ_0 (0.5 mM) reduction over 12 hours at 0.75 V_{RHE}. Light intensity: 100 mW cm^{-2} red light. Electrolyte solution: O_2-depleted Tris buffer (50 mM, pH 7.4).
